# The role of the dentist in the diagnosis and management of patients with oral mucosal diseases

**DOI:** 10.4317/medoral.24465

**Published:** 2021-01-04

**Authors:** Vinicius Coelho Carrard, Isaäc van der Waal

**Affiliations:** 1 Dept. of Oral Pathology, School of Dentistry. Federal University of Rio Grande do Sul, Porto Alegre, Brazil; 2 TelessaudeRS-UFRGS, Universidade Federal do Rio Grande do Sul, Porto Alegre, Rio Grande do Sul, Brazil; 3 Dept. of Oral and Maxillofacial Surgery/Pathology, VU medical centre. ACTA, Amsterdam, The Netherlands

## Abstract

Based on a few case reports of oral mucosal diseases a number of questions is raised about the role of dentists-general practitioners in the diagnostic procedure and management of patients with such diseases. For instance, are dentists prepared to prescribe topical corticosteroids and should dentists be taught how and when to take a biopsy? And how about palpation of the neck? A strong recommendation is made to take clinical pictures for proper documentation and, if needed, for telediagnostic procedures. Another issue relates to the communication between dentists and dental specialists when dealing with patients with oral diseases. In case of a patient suffering from burning mouth syndrome or any other type of chronic orofacial pain, the question is raised whether dentists-general practitioners are prepared to manage such patients. Furthermore, there is a call for structuring the collaboration between dentists-general practitioners and dental specialists, including oral and maxillofacial surgeons.

** Key words:**Oral diseases, management of oral diseases, intradisciplinary collaboration in dentistry.

## Introduction

Most dental consultations are related to caries and periodontal disease. These issues are usually properly addressed by general dentists, while more complex cases are referred to specialists in the different fields of dentistry, such as endodontologists, prosthodontists, implantologists and periodontologists. In many, if not most, parts of the world dentists are less well-prepared to diagnose and manage patients with oral mucosal diseases, resulting in professional diagnostic delay and erroneous treatments. On the other hand, redundant referrals to various specialists should be avoided. Such referrals often carry inconveniences for the patients, e.g. travel arrangements and absence from work, and may also result in an increase of healthcare expenses at large (1,2). In this respect the use of telediagnostics may be valuable.

The aim of the present treatise is to discuss the presumed level of expertise of dentists-general practitioners to properly diagnose and manage patients with oral diseases. For this reason, just a few examples of oral mucosal diseases will be presented.

## Discussion of a few selected mucosal lesions

- Case 1

During a routine dental visit a 17-year-old, otherwise healthy woman asked her dentist advice for recurrent, painful ulcerations in her mouth. She could not recall any precipitating events. She has been told already that these ulcers are called aphthous ulcers or recurrent aphthous stomatitis (RAS). Use of over-the counter drugs had not given her any relief, and she wondered whether the dentist could prescribe a more effective drug. The medical history was otherwise negative, and she did not smoke.

**Figure 1:**
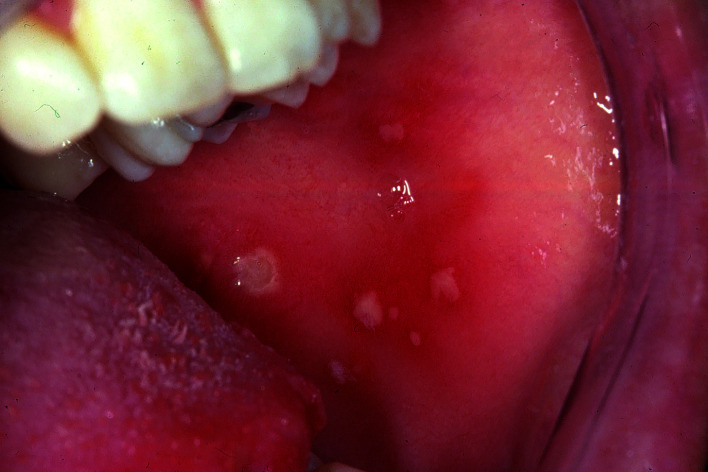
Multiple, painful recurrent ulcers in the buccal mucosa, more or less diagnostic of recurrent aphthous stomatitis.

At the time of her dental visit there were multiple pin-point seized ulcers present on the left buccal mucosa and a somewhat larger one on the right border of the tongue (Fig. 1). Based on the history and the clinical aspects, the dentist immediately arrived at the diagnosis of RAS.

Although there are a few conditions that may cause aphthouslike oral lesions, the dentist did not feel the need for blood examination or any other laboratory test, including the taking of a biopsy. Referral to a medical or dental specialist, such as an oral medicine specialist or an oral and maxillofacial surgeon, was not felt indicated either and the patient did not insist on such referral.

The dentist informed the patient, verbally and in writing, about the various aspects of RAS, including information about the limited possibilities to truly cure or prevent the disease. The patient was told that there may be some benefit from applying topical corticosteroids, e.g. triamcinolone acetonide 0.1%, particularly in the prodromal stage. In this case the patient rejected the use of topical corticosteroids. No special follow-up visit was scheduled.

In almost all cases the diagnosis RAS is a clinical one, not requiring any further tests. When the patient had consented in trying topical corticosteroids, the question would arise whether the dentist is prepared to handle such prescription. What dosage, when and how to use the drug (in the prodromal stage only?), for how long and what about the possible adverse side effects? Probably in many parts of the world dentists do not feel prepared to prescribe corticosteroids, not even for topical use, and, instead, prefer to refer the patient to a specialist. Another option might be the prescription of topical corticosteroids under the supervision of a specialist. The use of systemic corticosteroids is rarely indicated and, in our view, dentists should refrain from prescribing such drugs.

- Case 2

**Figure 2:**
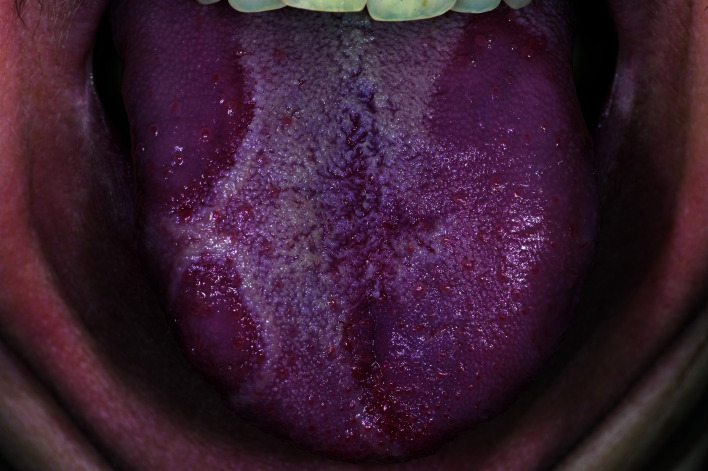
Geographic tongue; usually a clinical diagnosis.

A 60-year-old woman asked her dentist to have a look at her tongue. She experienced some recurrent irritation on the dorsal surface of the tongue during the last year. She was on medication for diabetes mellitus, smokes approximately 10 cigarettes a day and uses some four alcoholic consumptions per day. At examination, a somewhat unusual aspect of the dorsal surface of the tongue was noticed (Fig. 2) No other oral lesions were observed. What to do? Wait-and-see? For how long and at what intervals?

In the absence of a tentative diagnosis the latter wait-and-see suggestions does not seem to be appropriate. Management must, indeed, be based on a (tentative) diagnosis, even if shown to be wrong at a later stage. In this case the dentist decided to use a telediagnostic procedure.

Based on the clinical picture a definitive diagnosis of geographic tongue has been rendered by the consulted expert, making a referral for diagnostic purposes redundant. Given the benign diagnosis and the limitations of treatment there is no use in referring the patient for treatment or follow-up.

It is beyond the aim of the present text to further discuss the various aspects of geographic tongue, although it is well appreciated that the clinical aspect of geographic is not always that characteristic as being shown in textbooks.

It well recognized that making a 'telediagnosis' is not always possible or justified. In that case a 'physical' referral may be required. Needless to say that the results of 'teleconsulting communication' should always be included in the patients' record. As in case 1, proper patient information, preferably verbally and in writing, should be made available to the patient. One may even sit together with the patient to spend some time at Internet sites on this issue.

How about taking a biopsy in this case? Is the dentists trained to do so (3)? In general, we would advise not to perform a biopsy in the absence of a tentative diagnosis ('a biopsy should confirm the tentative diagnosis rather than detect the diagnosis'). If one is unable to come up with a tentative diagnosis, one may not be the proper person to perform a biopsy or to ask for any other (laboratory) studies. In such cases we encourage the taking of a clinical picture in order to be able to electronically ask a more experienced clinician, either a dentist or a dental specialist, for his or her advice. In daily practice, the taking of a biopsy in case of suspicion of geographic tongue is not indicated.

- Case 3

**Figure 3:**
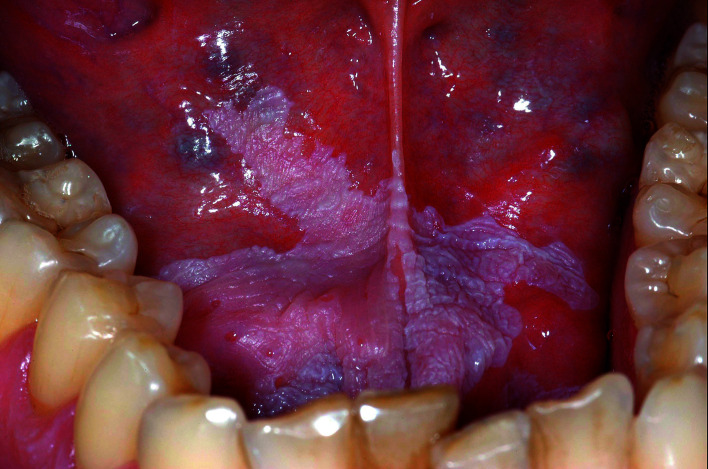
White changes in the floor of the mouth, indicative of leukoplakia.

During routine dental follow-up the dentist noticed a whitish lesion in the floor of the mouth in a 36-year-old man (Fig. 3).

The lesion was asymptomatic and the patient was not even aware of it. He was healthy otherwise and smoked some 15 cigarettes per day. He denied the use of alcohol. On palpation of the lesion no induration was present. No lesions elsewhere in the mouth were encountered. The dentist considered the diagnosis of leukoplakia, possibly caused by the smoking habits of the patient. How should the dentist proceed?

First, we strongly recommend to always take a clinical picture for proper documentation in the patients' record. If the clinical diagnosis of leukoplakia- a potentially malignant disease- is correct, the question arises whether a biopsy and possibly also treatment is indicated. Given a history of tobacco usage one might consider to strongly advise the patient to quit this habit and to evaluate the leukoplakia within a somewhat arbitrarily chosen period of three months. Such policy seems justified in case of an otherwise asymptomatic leukoplakia in which no induration is present. However, as in the previous case, we would recommend to first get advice from an expert, if possible by electronic communication. In this case, the expert will probably want to see the patient himself before being able to give advice about the management of the patient. In this case the expert, indeed, wanted to see the patient.

Since the patient was unable to quit smoking the specialist decided to perform CO2 laser evaporation after a preceding biopsy (which did not show unfavourable signs at histopathologic examination). Healing was uneventful and the dentists was asked to take-over the follow-up of the patient at intervals of 4-6 months.

A provisional clinical diagnosis of leukoplakia requires a thorough knowledge of all whitish, sometimes very rare lesions that may occur in the mouth, varying from aspirin burn, candidiasis, hairy leukoplakia, morsicatio, white sponge naevus, second stage syphilis and many more. Is a dentist-general practitioner supposed to be acquainted with all these lesions and disorders? Such assumption does not seem to be realistic. Instead, a provisional diagnosis of 'white mucosal lesion' seems quite appropriate, provided that a specialist is consulted for obtaining a final diagnosis and suggestions for the management of the patient.

Although CO2 laser evaporation is a rather safe procedure, its use by dentists-general practitioners is discouraged, unless one is properly trained to do so, including the issue of taking one or more biopsies before treatment.

Some dentists may be reluctant to accept the responsibility of follow-up visits because of lack of experience. In such event is the specialist should provide the dentist with guidelines what to look for during follow-up. Symptoms or changes of the size, the colour or the texture of the lesion are indications for a renewed diagnostic evaluation by the expert.

- Case 4

**Figure 4:**
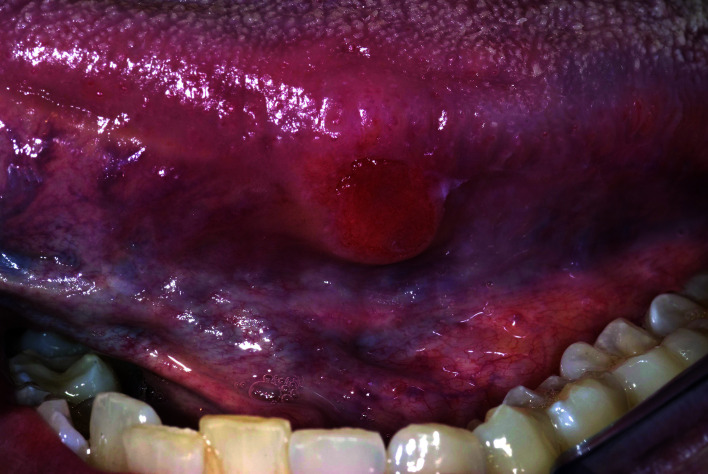
Slightly elevated and indurated ulcerative swelling of the border of the tongue, highly suspicious of squamous cell carcinoma.

A 32-year-old woman visited her dentist because of a painful lesion, present for some months, on the left border of the tongue (Fig. 4). She was a non-smoker; there was limited use of alcohol. There were no lesions elsewhere in the mouth. Because of the clinical aspect of the lesion, some induration on palpation and location on the border of the tongue, the dentist made a provisional diagnosis of a squamous cell carcinoma, in spite of the young age of the patient and the absence of aetiological factors. What would be the next step? Because of the rather strong suspicion of cancer a wait-and-see policy for 2-3-weeks did not seem appropriate. No need was felt for exfoliative cytology or a brush biopsy either, if such adjuncts may be helpful at all. The same applies to the use of other diagnostic aids, such as toluidine blue. The most logical next step in this case is the taking of a biopsy by an expert, preferably but not necessarily being an oncologic surgeon. From a dental background point of view an oral and maxillofacial surgeon trained in oncology would be the first choice.

May one expert from a dentist-general practitioner to arrive at a clinical diagnosis of squamous cell carcinoma or any other oral malignancy, also in view of the rare occurrence and the wide variety in which such cancers may present? Will it suffice to recommend to referring, physically or electronically, each patient who presents with an unidentified oral lesion, irrespective of the presence of symptoms and a 'benign' or 'malignant' appearance? And what about the possible presence of metastatic spread to one or more lymph nodes in the neck? Is the dentist-general practitioner properly trained to palpate the neck for suspicious lymph nodes? And what would be the relevance in this case to do so in the dental office?

- Case 5

A 62-year-old woman complains about a burning sensation of the tip of the tongue, being present every day and actually increasing during the day, for several months. She also complains about dryness of her mouth. She is not on medication. Oral examination, including inspection of the tongue, does not reveal any abnormalities. The dentist has never been confronted with a patient having this type of symptoms before. What should he or she do? The answer is to ask for advice from an expert, being an oral medicine specialist or an oral and maxillofacial surgeon.

Each dental expert in the field of oral diseases will immediately consider the diagnosis 'Burning mouth syndrome' (BMS). BMS is a rare disorder, mainly occurring in postmenopausal women. The aetiopathogenesis of this chronic pain syndrome is not well understood. In many cases patients also suffer from psychogenic disorders, not to be interpreted as the cause of BMS. It does not seem reasonable to expect from dentists-general practitioners to be acquainted with this syndrome and with the pharmacological and non-pharmacological (symptomatic) treatment modalities. Instead, the dentist is advised to frankly inform the patient of his or her lack of knowledge and expertise in this particular field, abstaining from incomplete or even erroneous information on this syndrome.

It seems obvious that this patient needs to be referred. But to whom? The family doctor, the otolaryngologist, the neurologist or perhaps the psychiatrist? In view of the dental background the recommended referral pattern is through an oral medicine specialist or an oral and maxillofacial surgeon. If needed, these specialists may arrange referrals to their medical colleagues or to a specialized, multidisciplinary pain clinic.

## Discussion and recommendations

Based on these somewhat at random chosen examples of oral diseases, that we could have extended with numerous other examples, e.g. vesiculobullous diseases and mucositis, several questions arise. Firstly, we recommend taking clinical pictures of oral mucosal lesions for proper documentation in the patients' records. Such photographs can also be used for telediagnostic procedures, if indicated (4). Secondly, dentists are strongly discouraged to leave a mucosal lesion undiagnosed, even when 'it looks benign', sometimes resulting in remarks like "Nothing to worry about". Some or perhaps the majority of the patients will accept such judgement, but others may insist on a precise diagnosis. Thirdly, the taking of biopsies or the use of other diagnostic aids by dentists-general practitioners seems only justified when dentists are properly trained to do so. Such training may be useful particularly when practising in remote areas.

Fourthly, dentists should be able to inform the patients about common oral diseases, while such information should be withheld in case of a rare or uncertain diagnosis, thereby avoiding incomplete or erroneous information. Another issue, number five, relates to palpation of the neck for the possible presence of enlarged lymph nodes. This subject is probably included in most undergraduate dental curricula in the world, but in reality, is not practised as such, and probably rightly so, by dentists- general practitioners. Palpation of the neck and interpretation of enlarged structures require extensive knowledge and experience in this field, including knowledge about the possible need for additional examinations (5). Sixthly, dentists should be able to properly and timely arrange referrals to specialists, in most cases being an oral medicine specialist or an oral and maxillofacial surgeon.

Seventhly, the question arises, related to case number 5 (Burning mouth syndrome) whether dentists-general practitioners are prepared to manage patients with chronic orofacial pain (6,7). Eightly, as demonstrated in the presently discussed patients, proper communication between dentists and dental specialists is a prerequisite for management of patients with oral diseases. At present, there are no international guidelines that structure the collaboration between dentists-general practitioners and dental specialists in case of diagnostic procedures and management strategies of oral mucosal diseases (8). Such guidelines may differ considerably in various parts of the world, depending on, among other things, the structure of the healthcare system, including the financial aspects. Recently, a preliminary study demonstrates that such initiative is valuable as an attempt to improve the referral process (9). Finally, we want to encourage to discuss the above items in Dental Associations, Associations of Oral Medicine, Associations of Oral and Maxillofacial Surgery and, of course, also in graduate and undergraduate programs in the dental field, including oral medicine and oral and maxillofacial surgery.
